# The Public Health Impact of a Ban on Flavored Cigars: A Decision-Theoretic Policy Framework

**DOI:** 10.1093/ntr/ntae173

**Published:** 2024-07-13

**Authors:** David T Levy, Christopher Cadham, Yoonseo Mok, Nargiz Travis, James H Buszkiewicz, Jihyoun Jeon, Nancy L Fleischer, Rafael Meza

**Affiliations:** Lombardi Comprehensive Cancer Center, Georgetown University, Washington DC, USA; Department of Health Managment and Policy, University of Michigan, Ann Arbor, MI, USA; BC Cancer Research Institute, University of British Columbia, Vancouver, Canada; Lombardi Comprehensive Cancer Center, Georgetown University, Washington DC, USA; Department of Epidemiology, University of Michigan, Ann Arbor, MI, USA; Department of Epidemiology, University of Michigan, Ann Arbor, MI, USA; Department of Epidemiology, University of Michigan, Ann Arbor, MI, USA; BC Cancer Research Institute, University of British Columbia, Vancouver, Canada

## Abstract

**Introduction:**

Half of adult cigar users report flavored cigars as their usual brand. The Food and Drug Administration proposed prohibiting “all characterizing flavors in cigars” and “menthol… in cigarettes.” We provide evidence on cigar and cigarette transitions and a framework to assess the impact of a U.S. flavored cigar ban.

**Aims and Methods:**

Using Population Assessment of Tobacco and Health survey waves 1–4, we estimated use patterns and annual transitions among flavored cigars, non-flavored cigars, cigarettes, and among adults aged 18–34 and aged ≥35. We also consider electronic nicotine delivery systems (ENDS)-related transitions. We developed a decision-theoretic framework for examining the impact of a flavored cigar ban alone, and the impact of a flavored cigar with a menthol cigarette ban with and without a non-tobacco flavored ENDS ban.

**Results:**

Cigar users exhibited less stable use than cigarette users, with a large portion of cigar users switching to cigarette use each year. Past studies provide limited information on transitions between cigar and ENDS use. Our policy framework suggests that imposing a flavored cigar ban alone may be partially undermined by the substitution of menthol cigarettes for flavored cigars. While adding a menthol cigarette to a flavored cigar ban is expected to improve public health, a simultaneously implemented ENDS may offset some of the gains.

**Conclusions:**

Our analysis suggests the information necessary to gauge the public health impact of a cigar flavor ban alone and with flavor bans on cigarettes and ENDS. Further research is needed on ENDS vis-a’-vis cigar use, and the impact of enforcement and non-flavor-related policies on flavor ban effectiveness.

**Implications:**

Unlike menthol cigarette use and menthol bans, flavored cigar use and flavored cigar bans have received minimal attention. Transitions from cigars, especially dual and flavored use, are generally common compared to cigarettes. Our policy framework suggests important public health impacts. A flavored cigar ban absent a menthol cigarette ban may be partially undermined by the substitution of menthol cigarettes for flavored cigars. Adding a menthol cigarette ban is expected to offset such substitution and improve public health. However, simultaneously adding an ENDS with a flavored cigar and menthol cigarette ban may reduce the public health impact of a menthol cigarette and cigar flavor ban since flavored cigar users would be less able to substitute a lower-risk alternative.

## Introduction

The use of filtered cigars, cigarillos, and traditional premium or non-premium cigars is associated with increased all-cause, cardiovascular and respiratory disease mortality risk.^1–5^ While the prevalence and average annual per capita U.S. sales for cigarettes and cigars all declined from 2000 to 2020, cigars experienced a more gradual decline.^6^ Among youth and young adults, cigar use has been declining at a slower rate than cigarette use,^7^ with last-30-day cigar use among high schoolers equaling or surpassing cigarette use in 2022^8^ and 2023.^9^ In 2021, 3.5% of all U.S. adults and 4.9% of those who are aged 25–44 smoked cigars.^10^

A premium cigar is typically handmade, large, filled with at least 50 percent natural long-leaf filler tobacco, wrapped in whole-leaf tobacco, and without filters or tips.^5^ Flavored cigars are limited to “non-premium” cigars, including filtered cigars, cigarillos, and large traditional non-premium cigars.^11–14^ From 2009 to 2020, flavored cigar sales increased by 3.5% annually.^15^ Half of the adults and more than half of the youth who smoked non-premium cigars reported that their usual brand is flavored.^5,16–18^ Flavored cigar use is highest among vulnerable populations, including youth and young adults, non-Hispanic Black and Hispanic adults, and those of low SES.^18–22^ Youth^23^ and adults^5,16^ claim that flavors are an integral reason for using cigars.

Flavor bans are a popular measure for combating tobacco use. The U.S. Food and Drug Administration (FDA) banned non-menthol cigarette flavors in 2009. Many local jurisdictions^24–27^ and states^28^ have since implemented non-cigarette tobacco flavor bans, in some cases including cigars. In 2022, the FDA proposed separate standards prohibiting “all characterizing flavors in cigars”^29^ and “prohibiting menthol as a characterizing flavor in cigarettes,”^30^ emphasizing the role of flavors in attracting youth tobacco use and in increasing tobacco-related diseases.^31^ Rostron et al.^32^ estimated that a U.S. flavored cigar ban would annually yield 800 fewer cigar-attributable deaths.

While menthol cigarette use and its ban have received considerable attention,^33–35^ the impact of a flavored cigar use and the impact of a flavored cigar ban has seen minimal attention. Because flavored cigar use is mainly limited to filtered cigars, cigarillos, and large traditional non-premium cigars,^11–14^ and premium cigar users are less likely to smoke cigarettes,^11^ we focus on non-premium cigar use. First, we examine use rates and transitions between flavored cigars, non-flavored cigars, and cigarettes without a general flavored cigar or menthol cigarette ban. We separately consider potential transitions to electronic nicotine delivery systems (ENDS, aka e-cigarettes). Next, we develop a policy framework for examining the public health impact of a flavored cigar ban under three scenarios: a flavored cigar ban alone, a flavored cigar and menthol cigarette ban, and a ban on flavored cigars, menthol cigarettes, and non-tobacco-flavored ENDS. We conclude by discussing gaps in our knowledge, including ENDS vis-a’-vis cigar use, and the potential role of enforcement and non-flavor-related policies.

## Use of Flavored Cigars, Non-flavored Cigars, and Cigarettes

### Methods

We estimated prevalence and transition rates from the nationally representative Population Assessment of Tobacco and Health (PATH) Survey for waves 1–4, occurring from 2013 to 2017 before most states or cities had implemented a cigar flavor, menthol cigarette, or ENDS flavor ban. We apply the same approach as Jeon et al.,^13^ but excluding premium cigar users. Premium cigar use was distinguished by brand name, and by price when brand name was not available.^5,13^

Non-premium cigar users include those: (1) who reported ever “fairly regular” use of traditional non-premium cigars, cigarillos, or filtered cigars and (2) reported currently using one or more of those cigar products on some days or every day. In attempting to develop a public health-oriented measure of regular use, we restricted current established cigar use to those reporting use ≥ 5 days of the past 30 days summed over all cigar products. Cigar use in <5 of the past 30 days is included as non-current/infrequent use. Current established cigarette use was defined as smoking at least 100 cigarettes lifetime and currently smoking cigarettes every day or some days. Current dual use of cigars and cigarettes was defined as meeting both the cigarette and cigar criteria for regular use. We distinguished flavored from non-flavored use based on cigar users reports regarding whether their regular/last brand was flavored.

To focus primarily on exclusive versus dual use and flavored versus non-flavored cigar use, we do not distinguish transitions for specific cigar types, for example, filtered cigars versus cigarillos. Further breakdown of categories by cigar type would have led to small samples (ie, <5) for many of the transitions and, thus, not sufficiently reliable estimates.^36^ Due to differences in use patterns^37–39^ and transitions by exclusive and dual cigarette and cigar users,^40,41^ those reporting exclusive blunt (the hollowed cigar, cigarillo, or filtered cigar shell used for marijuana) use were not included as cigar users. They were instead classified as non-current cigar users.

Transition rates are measured in relative terms as the average annual percent of users that transition between product categories. We estimated prevalence and 1-year transition rates by averaging over the first four waves (2013 to 2017).

### Results

Table 1 presents use and transition rates for individuals aged 18–34, the ages when combustible use often becomes established.^5,17,42^ Initial non-premium cigar prevalence (first column) was 2.2% in total (ranging from 0.4% to 0.7% by category) compared to 20% cigarette prevalence. Regarding transitions, 31% of exclusive non-flavored versus 22% of exclusive flavored and 24% of dual non-flavored versus 14% of dual flavored cigar users remained in the same cigar use category, compared to 83% of exclusive cigarette users (yellow shaded diagonal). A large percentage of non-flavored (64%) and flavored (56%) dual cigar users while only 8% of non-flavored and 11% of flavored exclusive cigar users transitioned to exclusive cigarette use (shaded gray). Exclusive cigarette users rarely (≤1.5%) transitioned to exclusive or dual cigar use (shaded green). Transitions between flavored and non-flavored exclusive cigar use and between non-flavored and flavored dual use ranged from 7-9% (shaded pink). About 31% of exclusive cigar users compared to 14% of cigarette and 11% of dual cigar users quit all combustible use (shaded orange).

**Table 1.** The Average Prevalence and Transition Rates of Cigarette and Cigar Users, 5+ of Past-30 Days for Current Cigar Excluding Premium Cigar Users, PATH Waves 1–4, Male and Female, Ages 18–34

	Year 2 never regular (62.3%)	Year 2 non-current/ infrequent (15.4%)	Year 2 exclusive cigar, non-flavor (0.4%)	Year 2 exclusive cigar, flavor (0.3%)	Year 2 exclusive cigarette (20.6%)	Year 2 dual cigar, non-flavor (0.4%)	Year 2 dual cigar, flavor (0.6%)
Year 1 never regular (64.7%)	96.4%	1.8%	0.1%	0.1%	1.5%	0.0%	*
Year 1 non-current/ infrequent (13.1%)	—	82.8%	0.7%	0.8%	15.0%	0.2%	0.4%
Year 1 exclusive cigar, non-flavor (0.4%)	—	46.6%	31.1%	9.1%	7.8%	3.6%	1.7%
Year 1 exclusive cigar, flavor (0.5%)	—	50.5%	7.4%	22.2%	11.1%	2.7%	6.1%
Year 1 exclusive cigarette (20.2%)	—	14.0%	0.1%	0.1%	83.4%	1.0%	1.5%
Year 1 dual cigar, non-flavor (0.4%)	—	11.4%	2.8%	*	63.8%	13.8%	7.9%
Year 1 dual cigar, flavor (0.7%)	—	11.4%	0.8%	1.6%	55.9%	6.7%	23.6%

Y1 = year 1, Y2 = year 2. Non-current = former cigarette or non-premium cigar user; * not distinguishable (<5 observations).

Table 2 shows an initial overall cigar use prevalence of those aged 35+ of 1.6%, with slightly higher percentages of dual than exclusive users. Of cigar users, 34%–49% (highest among exclusive non-flavored cigar users) remained in the same use category, while 89% of exclusive cigarette users remained cigarette users. About 4% of non-flavored and flavored exclusive cigar users transitioned to exclusive cigarette use (shaded gray), while 37% of non-flavored and 40% of flavored dual users transitioned to exclusive cigarette use. Exclusive cigarette users mostly transitioned to flavored (0.9%) or non-flavored (0.8%) dual use (shaded green). From 6% to 9% transitioned between flavored and non-flavored exclusive cigar use, and 8% to 11% transitioned between flavored and non-flavored dual use (shaded pink). Exclusive cigar (32%) were more likely than exclusive cigarette (9%) users to quit all combustible product use, while 4% of dual non-flavored and 11% of dual flavored cigar users quit all combustible use (shaded orange). Compared to those aged 18–34, cigar users aged 35+ showed greater stability in maintaining the same category of cigar use and lower rates of quitting all combustible use, and had lower rates (although still 37%–40%) transitioning from dual use to exclusive cigarette use.

**Table 2.** The Average Prevalence and Transition Rates of Cigarette and Cigar Users, 5+ of Past-30 Days for Current Cigars Excluding Premium Cigar Users, PATH Waves 1–4, Male and Female, Ages 35 and Above

	Year 2 never regular (52.9%)	Year 2 non-current/infrequent (29.3%)	Year 2 exclusive cigar, non-flavor (0.3%)	Year 2 exclusive cigar, flavor (0.2%)	Year 2 exclusive cigarette (16.1%)	Year 2 dual cigar, non-flavor (0.3%)	Year 2 dual cigar, flavor (0.4%)
Year 1 never regular (54.8%)	96.5%	2.5%	0.1%	0.0%	0.9%	0.0%	*
Year 1 non-current/infrequent (28.1%)	—	96.0%	0.2%	0.1%	3.5%	0.0%	0.1%
Year 1 exclusive cigar, non-flavor (0.3%)	—	31.8%	48.9%	6.0%	4.4%	7.0%	1.8%
Year 1 exclusive cigar, flavor (0.2%)	—	31.0%	8.5%	39.7%	3.9%	2.0%	14.9%
Year 1 exclusive cigarette (16.0%)	—	9.0%	0.0%	0.1%	89.3%	0.8%	0.9%
Year 1 dual cigar, non-flavor (0.3%)	—	4.0%	7.9%	*	36.9%	38.5%	11.4%
Year 1 dual cigar, flavor (0.4%)	—	11.0%	1.1%	5.6%	40.3%	7.9%	34.1%

Y1 = year 1, Y2 = year 2. Non-current = former cigarette or non-premium cigar user; * not distinguishable (<5 observations).

## Cigars, Cigarettes and Ends Use

The above analysis did not consider transitions involving ENDS. One study found that ENDS use often precedes youth and young adult cigar use^43^ and another study found that ENDS and cigar use precedes youth cigarette use.^44^ At the same time, studies have found that youth cigarette use dramatically fell as ENDS use increased.^45–49^ While trends in ENDS and youth cigar use have not been similarly considered, the slower decline in youth cigar than cigarette use^7–9^ may be due to less substitution of ENDS for cigar than cigarette use. Studies also indicate that frequent ENDS use improves cessation from cigarette use.^50,51^ In addition, dual users of ENDS and menthol cigarettes were more likely to use menthol/mint-flavored ENDS and switch completely to ENDS^52^ and were more likely to quit smoking than dual ENDS and non-menthol cigarette users.^53,54^ We found no studies examining the relationship of ENDS use to flavored and non-flavored cigar cessation, but both ENDS and non-premium cigar users cite flavors as a primary reason for their use.^23^

## Framework to Analyze the Public Health Impact of a Flavored Cigar Ban

As in previous public health impact analyses of ENDS use^55^ and heated tobacco product use,^56^ we apply a decision-theoretic framework to consider the potential implications of flavored cigar bans. Our policy-oriented framework considers the public health impact of the following scenarios: (1) a flavored cigar ban alone relative to no bans, (2) a flavored cigar and menthol cigarette ban relative to a flavored cigar ban alone, and (3) a flavored cigar, menthol cigarette, and flavored ENDS ban relative to a flavored cigar and menthol cigarette ban.

We focus on the impact of a flavored cigar ban on exclusive and dual flavored cigar users. We present the impact on overall adult use, but suggest differences in impacts between younger (eg, ages 18–34) and older (eg, age 35+) users. To simplify the analysis, we assume that exclusive ENDS use imposes lower risks while dual use has the same health risks as exclusive cigarette use.^57,58^

In each figure below, the boxes on the far right represent potential final outcomes. Public health impacts for final outcomes are colored green for gains and pink for losses, with darker shades indicating greater potential impact (eg, darker green indicates a larger expected transition than lighter green).

### Flavored Cigar Ban Alone

**Figure 1.**
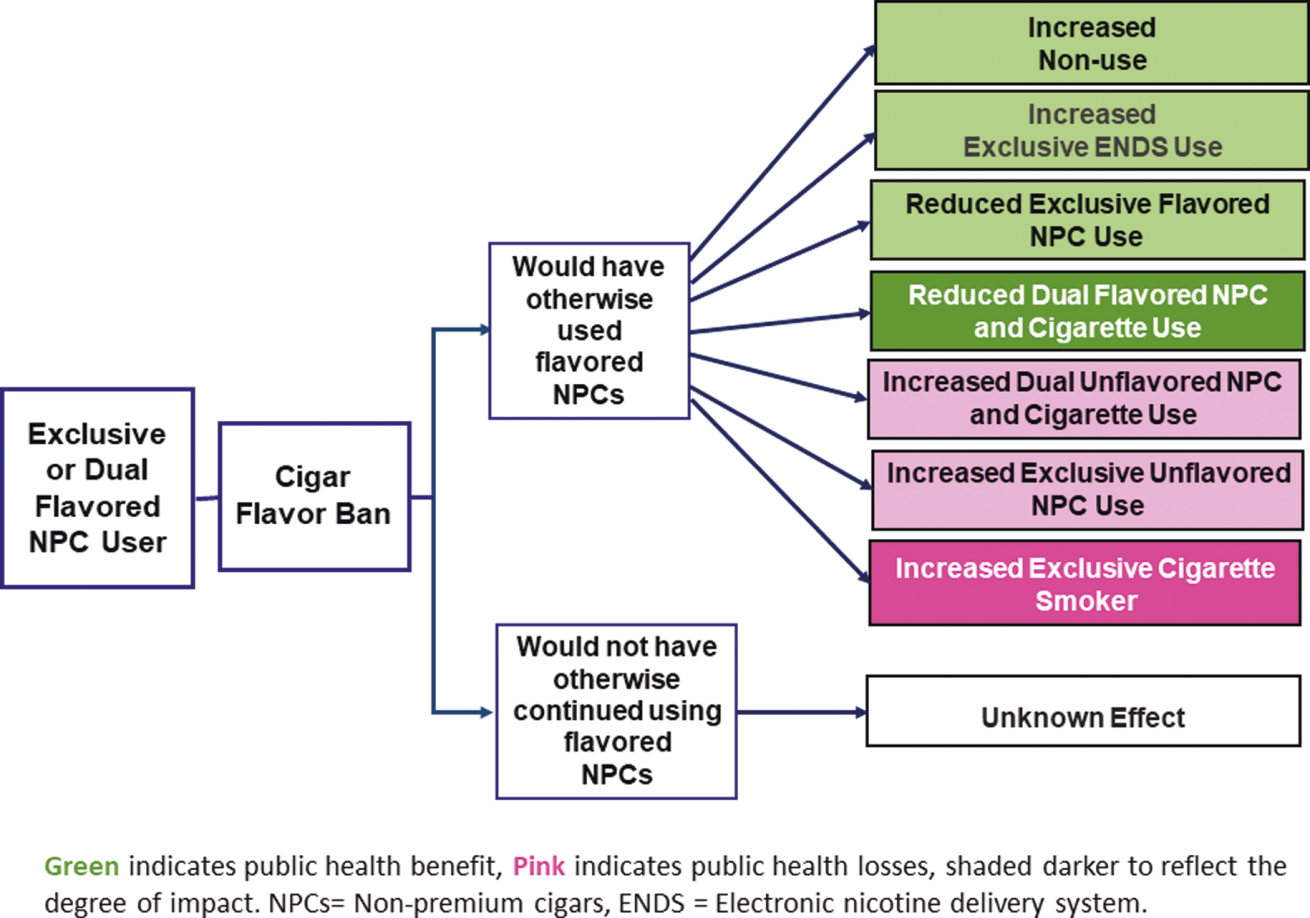
The public health impact of a flavored cigar ban alone on the use of cigarettes, cigars, ENDS, and no use by flavored cigar users.

Figure 1 shows the potential impact of a flavored cigar ban alone relative to no flavor bans. The flavored cigar ban may yield public health gains as exclusive and dual cigar users switch to exclusive ENDS use or quit all nicotine product use (possibly with the temporary aid of ENDS). However, some flavored cigar users may remain flavored cigar users, for example, via illegal purchase or adding their own flavors to non-flavored cigars and some dual cigar users switch to exclusive cigarettes, especially to menthol cigarette uses. Transitions from flavored cigars to non-flavored cigars may also yield offsetting losses if non-flavored cigar or cigarette users are less likely to quit, for example, if those who were flavored cigar users would have instead switched to flavored ENDS.

While our transition analysis in the previous section was conducted from 2013 to 2017 before most states banned flavored cigars, menthol cigarettes and flavored ENDS, the results are consistent with the impacts suggested by our framework analysis of a flavored cigar ban alone. A large percentage of dual-flavored cigar users (46% aged 18–34 and 40% aged 35+) transitioned to exclusive cigarette use, suggesting the potential for increases in initiation and switching into cigarette use under a cigar flavor ban. A smaller percentage of flavored exclusive cigar users (7%–9%) switched to non-flavored use, suggesting lower switching rates among this group. The patterns indicated by Figure 1 are also consistent with studies of a (sole) ban on flavored little cigars in Canada, which found reduced flavored cigar sales^59^ and reduced youth and young adult cigar use.^60^ A discrete choice experiment involving flavored cigar users also suggested that a flavored cigar ban alone would encourage cessation from flavored and overall cigar use.^41^ Furthermore, first cigar use that involved flavored cigars is associated with later regular cigar use.^61–63^ In addition, the 2009 U.S. flavored cigarette ban was followed by increased flavored cigar unit sales^64^ and increased youth cigar use,^65^ indicative of substitution between cigarettes and cigars. While we consider the impact of a cigar flavor ban on flavored cigar users, there may be an impact on non-flavored cigar users. For example, our transition analysis suggests that 7%–11% transition from non-flavored to flavored cigar use each year absent a cigar flavor ban. The likelihood of transitioning to cigarette, ENDS or no use under a flavor ban may differ if a non-flavored cigar user would have switched to flavored cigars absent the ban. However, a cigar flavor ban may discourage non-flavored cigar users from even temporarily switching to flavored cigar use. Evidence on transitions between flavored cigar and ENDS use is lacking.

### A Flavored Cigar Ban With a Menthol Cigarette Ban

**Figure 2.**
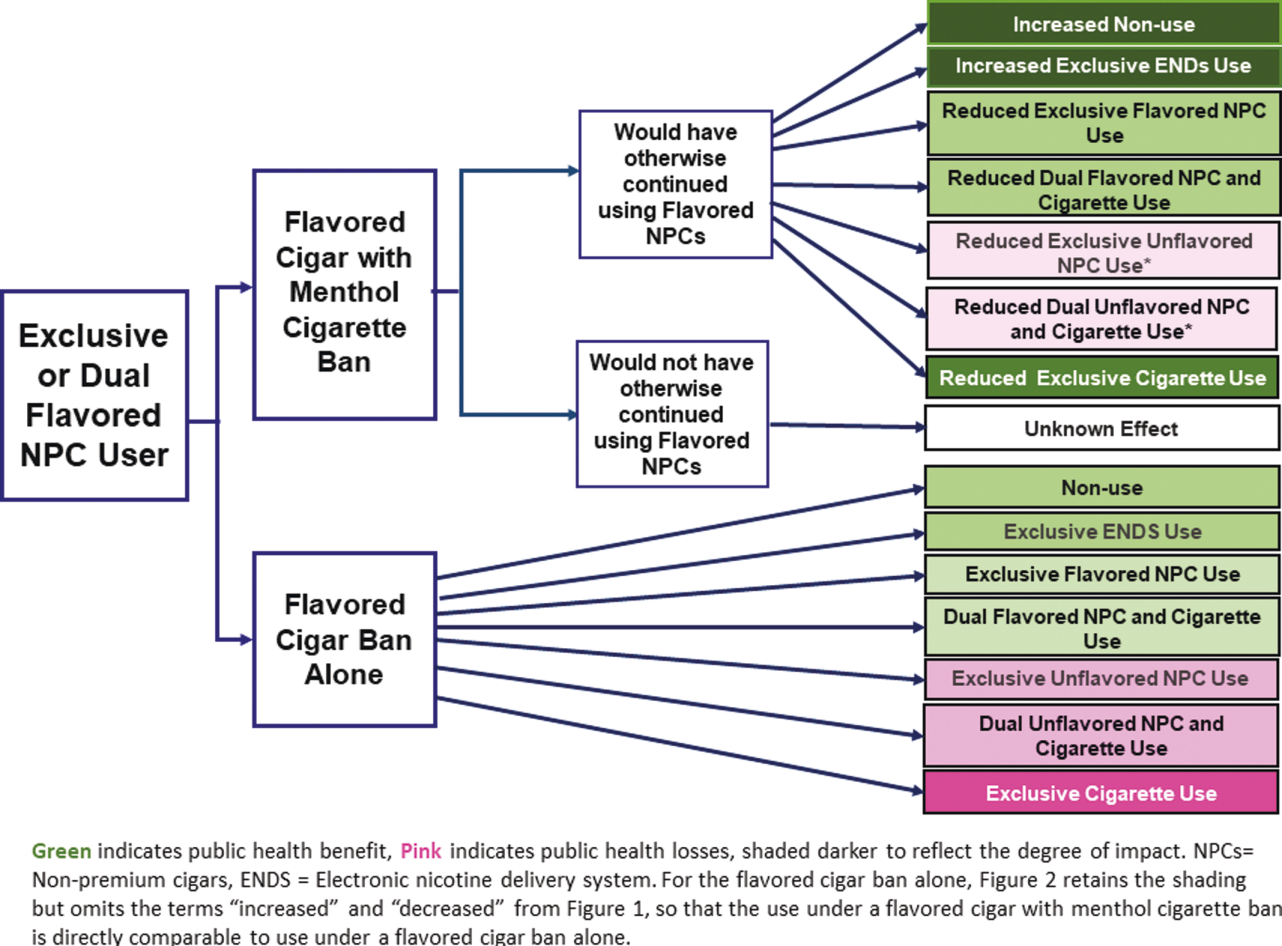
The public health impact of a flavored cigar with menthol ban on cigarettes, cigar, and ENDS or no use by flavored cigar users.

Figure 2 shows the impact of a menthol cigarette ban with an accompanying cigar flavor ban (top right rows) compared to a flavored cigar ban alone (bottom right rows). As in Figure 1, Figure 2 shows that a flavored cigar ban alone is likely to lead to less flavored cigar use, and more ENDS use and non-tobacco use, but increased non-flavored cigar and cigarette use. For a flavored cigar ban alone, Figure 2 retains the shading but omits the terms “increased” and “decreased” for a cigar flavor ban alone in order to compare a flavored cigar with menthol cigarette ban directly to a flavored cigar ban alone. We expect that a flavored cigar with menthol cigarette ban would improve public health compared to a flavor cigar ban alone, as those who would have smoked menthol cigarettes under a flavored cigar ban alone (darker green) switch to ENDS or nonuse (darker shaded green). In addition, fewer flavored cigar users (darker green) may switch to dual non-flavored cigar use (lighter pink), absent menthol-flavored cigarettes.

Our transition analysis indicates that a relatively large percentage of exclusive smokers (14% aged 18–34 and 9% aged 35+) quit cigarette use during the pre-flavor ban period, while a large percentage of dual flavored cigar users (60% aged 18–34 and 40% aged 35+) transitioned to exclusive cigarette use. These transitions indicate that smokers may be more likely to quit and dual flavored cigar users may be less likely to switch to cigarette use when a menthol cigarette ban is implemented with a cigar flavor ban. With increasing ENDS use,^7,66,67^ cigarette smokers, especially those aged 18–34, may also be more likely to transition to ENDS. In estimating the direct impact of a menthol cigarette ban alone compared to a flavored cigar and a menthol cigarette ban, a panel of experts^34^ predicted that those aged 35–54 who currently smoke menthol cigarettes would show a 7% net increase in combustible (cigarette and cigar) use following a menthol cigarette ban relative to a ban on both menthol cigarettes and flavored cigars. In addition, they predicted that 50% of those no longer smoking would switch to ENDS or smokeless tobacco.^34^ Applying the expert analysis, a simulation study found that a menthol cigarette ban with a flavored cigar ban^35^ yields substantial public health gains primarily through reduced cigarette use. Studies also support expert views. One study showed increased clove cigar use following the 2009 U.S. flavored cigarette ban.^68^ Cigar sales declined and ENDS sales increased after Minnesota extended its general flavor ban to menthol and mint flavors.^69^ Studies^70-75^ also indicate that ENDS provides an important alternative to combustibles under a menthol cigarette ban.

### A Flavored Cigar and Menthol Cigarette Ban With Non-tobacco Flavored ENDS Ban

**Figure 3.**
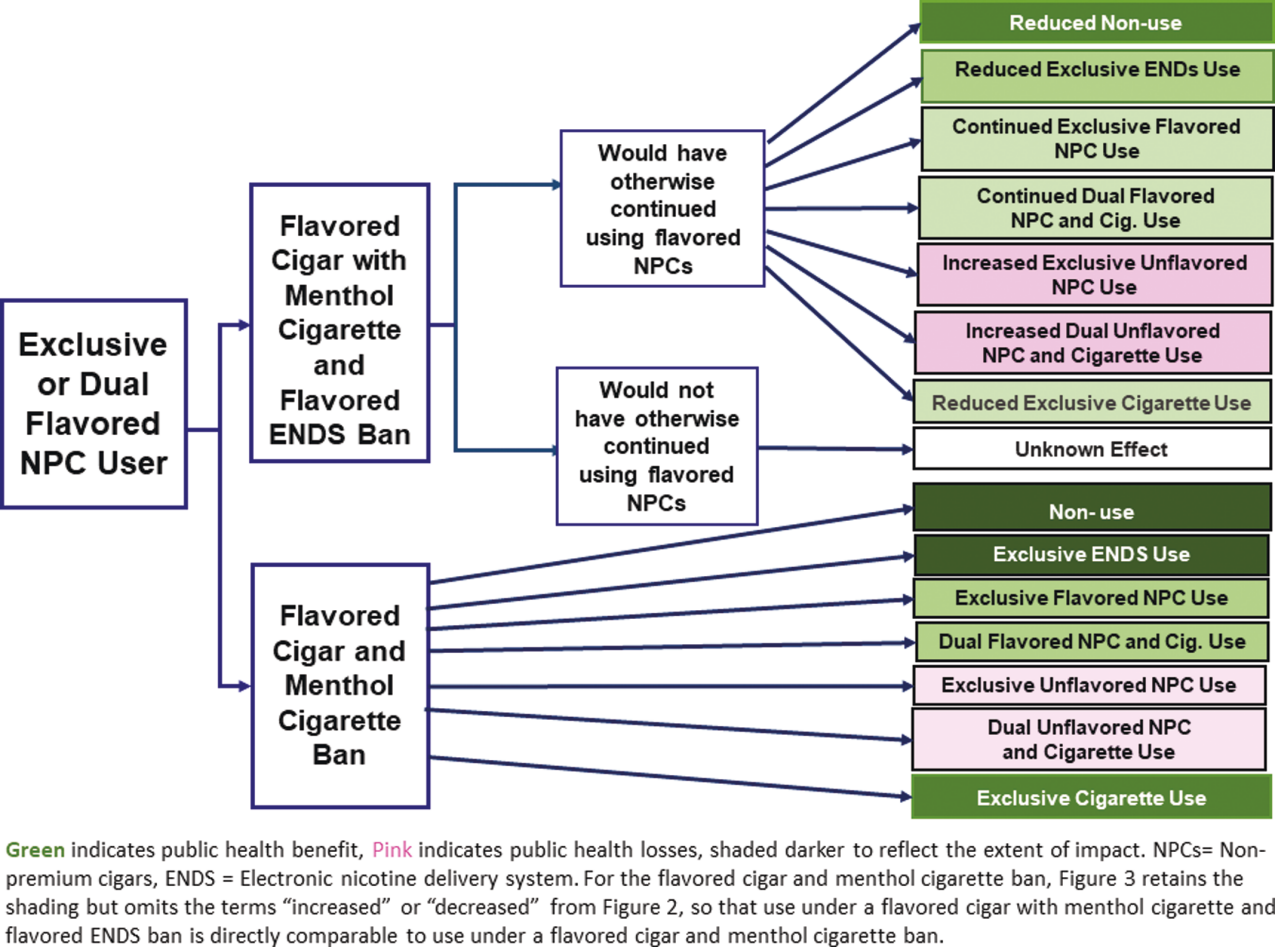
The public health impact of an ENDS flavored ban with menthol cigarette and flavored cigar ban on cigarettes, cigar, and ENDS or no use by flavored cigar users.

Figure 3 shows the impact of an ENDS non-tobacco flavor ban in conjunction with a flavored cigar and menthol cigarette ban compared to just a menthol cigarette and flavored cigar ban. Figure 3 posits that adding an ENDS flavor ban to a menthol cigarette and flavored cigar ban may yield public health losses due to the lack of flavored ENDS as a substitute for flavored cigars and menthol cigarettes. The losses occur as dual and exclusive flavored cigar users transition to exclusive and dual non-flavored cigar use (darker pink) or continue as (illicit) flavored cigar users instead of transitioning to ENDS use (lighter green) as an improved health alternative. Public health losses may also occur as fewer cigar and cigarette users become ENDS users or use ENDS to transition to no use (lighter green).

Our transition analysis did not consider ENDS use, but our review of the ENDS literature indicates that ENDS is associated with cessation from cigarettes and that menthol cigarette users were more likely to use ENDS and more likely to quit using ENDS. While evidence is mixed, studies have also found increased cigarette use following an ENDS flavor ban, implying that ENDS is a substitute for cigarettes.^76,77^ Several studies have examined the impact of a cigar flavor ban. A study of four states that implemented a flavored cigar ban preceded by an ENDS flavor ban^28^ found an average 25% reduction in flavored cigar sales without corresponding increases in non-flavored cigar sales. A comparison^78^ of the Massachusetts ENDS and combustibles flavor ban to the New Jersey ban on only flavored ENDS found reduced use of all tobacco and ENDS products in Massachusetts but increased flavored cigar and decreased menthol ENDS use in New Jersey.^78^ Studies of a Providence non-cigarette flavor ban have also found reduced flavored cigar sales^79^ and reduced youth cigar use.^24^ Comprehensive bans on all flavored products, including flavored cigars, find reductions in youth^26,80^ and young adult^81^ flavored tobacco use, and cigar sales.^25,82^ Because states have generally implemented ENDS flavor bans before menthol cigarette and flavored cigar bans, the specific impact of the flavored cigar flavor ban is unclear, for example, the ENDS flavor ban may increase cigar use rather than providing an offsetting low-risk alternative. Because of the information on ENDS use by cigar users is limited and potential substitution patterns are complex, our analysis of the impact of an ENDS flavor ban in conjunction with a flavored cigar and menthol cigarette ban is more tentiative than our policy analyses above.

## Discussion

Our analysis of pre-flavor ban (2013–2017) data indicates that cigar users maintained less stable use patterns than cigarette users, consistent with a recent study on the stability of cigar use.^83^ While substitution between flavored and non-flavored cigar use was limited, a large percentage of cigar users, especially dual users, switched to exclusive cigarette use, consistent with findings that cigars and cigarettes are close substitutes.64^,84-88^ Dual users were also less likely to quit both products than exclusive cigar or cigarette users, consistent with findings of less cessation among polytobacco users.^83,89–91^

Our policy framework suggests that a flavored cigar ban alone is likely to have a limited public health impact relative to a menthol cigarette and cigar flavor ban, primarily due to the substitutability of cigarettes and cigars. However, if implemented with a menthol cigarette ban, a flavored cigar ban would have greater potential to reduce overall combustible use and thereby improve public health. While not explicitly distinguished, our analysis similarly implies reduced public health benefits from a menthol cigarette ban without a flavored cigar ban. We also expect reduced public health gains if a menthol cigarette and flavored cigar ban is implemented with an ENDS flavor ban, because those who smoke cigars (and cigarettes) have less options to substitute a lower-risk alternative. In particular, menthol and mint-flavored ENDS restrictions are likely to reduce the effectiveness of a menthol cigarette ban. We focused on current flavored cigar users, but similar impacts are expected for those who would have initiated flavored cigar use. Further research is needed to evaluate transitions involving cigars under newly implemented flavor bans.

Due to limited sample size, we did not distinguish between types of cigar users, for example, filtered cigars, cigarillos and traditional non-premium cigars, when estimating transitions. Both Corey et al. (2013 PATH)^92^ and Jeon et al. (2017 PATH)^13^ find a higher prevalence of cigarillo than filter-cigar use in younger age groups. Thus, our estimated transitions for ages 18–34 likely reflect greater cigarillo use than the transitions for those aged 35+. Corey et al.^92^ find similar percentages of filtered-cigar and cigarillo users who regularly use flavored cigars (both near 60%). However, filtered-cigars are more likely to act as a substitute for cigarettes.^86^ Nevertheless, variations by age, intensity of use at different ages, and flavor type merit further attention, especially in distinguishing the impact of flavor bans.

Our transition and policy analysis also does not incorporate blunt use. Blunt use is prevalent among young cigar users,^37–39^ particularly African Americans.^93^ Less expensive flavored cigar/cigarillo brands are almost exclusively used as blunts.^37,38,94,95^ Studies exploring the reactions of cigar users to a hypothetical cigar flavor ban suggest that blunt users may be less likely to quit cigar (blunt) use and those who quit tend to switch to marijuana products.^40,41^ Consequently, blunt use may depend on local marijuana as well as flavored cigar ban policies. Indeed, the market share of a popular Swisher cigarillo brand used for blunts was higher in areas that legalized marijuana in the United States.^96,97^ Further research is warranted on blunt use and on policies to reduce co-use of cannabis and cigars.

While we considered ENDS as a potential harm-reducing alternative, studies are needed on the relationship of ENDS to cigar use in terms of initiation by youth and young adults and of cessation among adults. Evaluation of the impacts of flavored cigar, menthol cigarette, and ENDS flavor bans is further complicated by the presence of other reduced-risk nicotine delivery products. Smokeless tobacco has traditionally included non-tobacco flavors.^98^ While the FDA has restricted the authorized availability of flavored ENDS through the PMTA process,^99^ nicotine pouches^100,101^ and heated tobacco products^102^ are also available in flavors. Research is needed to consider whether these potentially reduced-harm products tend to serve as substitutes for or are used together with cigars (ie, dual use).

We focused on the U.S. population as a whole. Flavored cigar use is highest among non-Hispanic Black and Hispanic adults and those with low educational attainment or living in low-income households.^18–22^ Between 2002 and 2019, cigar use fell from 5.4% to 4.3% among non-Hispanic Whites and 5.2% to 3.3% among Hispanics, but increased among non-Hispanic Blacks (7.1% to 8.7%).^6,10^ Consequently, adopting a flavored cigar ban raises key equity issues.^103,104^ The impact of industry marketing and flavor ban compliance in racial and SES neighborhoods merits special attention.^105^

Our analysis did not explicitly consider the role of enforcement and compliance. The FDA Scientific Assessment of the Impact of Flavors in Cigar Products^106^ cites the need for improved compliance. Previous policy evaluations of flavored product bans depend on existing compliance levels. A federal ban would likely reduce the number of consumers crossing borders to obtain flavored products.^106^ Studies^107–110^ also find that flavored tobacco products are still available despite state and local flavored combustible bans. Much of the focus in past ban studies has been on mass-market retail, but cigars, cigarettes, and especially ENDS are available from a variety of sources, for example, tobacco shops, the internet, and vape shops.^111^ Future flavor ban studies should consider differences in compliance across these supply sources. Although not considered in our analysis, studies should evaluate the intensity (eg, quantity per day or days per month) of exclusive and dual products when gauging the public health impact of policies.^1–5^

Industry reactions to flavored cigar bans also merit attention. The largest U.S. cigar company is Swisher (40%+ market share) followed by Altria (Black and Mild) with a 30% market share.^112^ As a highly concentrated industry, cigar companies may engage in anticompetitive behavior to promote their aims. In reaction to a cigar ban, they may encourage switching from flavored to non-flavored cigars, encourage dual use, and discourage transitions to exclusive ENDS use. In addition, Farley et al.^113^ found that the tobacco industry circumvented flavor bans using flavor-characterizing names to introduce flavors without specifying the name. Cigar companies may also circumvent bans by encouraging the self-administration of flavor additives, as cigarette companies have done with menthol.^114–117^ Altria, the largest U.S. cigarette company, may also encourage substitution towards cigarettes. It will be essential to monitor industry marketing strategies, including relative pricing of cigars and cigarettes, price discounting, and advertising strategies. Enforcement will be critical in determining the impact of a cigar flavor ban.^118^

Studies should also consider the impact of flavor restriction policies in the context of the larger tobacco control policy space. The presence of non-flavor-oriented policies, such as Tobacco 21, higher cigarette and cigar taxes, and retail display bans, may affect the impact of a cigar flavor ban, for example, acting synergistically to increase the effectiveness of a cigar flavor ban.^79^ While our analysis focused on a federal U.S. flavored cigar ban, researchers could apply the same analysis at the state level and to other countries where cigar use is prevalent.

In conclusion, our policy framework indicates potential transitions under a flavored cigar ban, and the potential role of concomitant menthol cigarette and ENDS flavor bans. Menthol cigarette bans, in particular, are expected to play a crucial role by reducing access to a likely substitute for flavored cigars. The impact of an ENDS flavor ban is less clear, with its impact further complicated by the potential role of other reduced-harm nicotine delivery products. By focusing on the complexity of potential transitions that arise in public health analyses of tobacco flavor restrictions, we distinguish the following areas that merit particular attention: (1) differential impacts by race/ethnicity and socioeconomic status, (2) transitions involving cigarettes, flavored and non-flavored cigars and dual and exclusive cigar use, (3) the role of ENDS and other reduced-harm nicotine delivery products in cigar transitions, and (4) the role of flavor ban compliance and of other non-flavor tobacco control policies. While uncertainty remains about product use patterns and the role of new products, existing studies provide vital information on the potential public health impact of cigar flavor bans and limitations of such a policy if applied inconsistently across high-risk product classes.

## Supplementary material

Supplementary material is available at *Nicotine and Tobacco Research* online.

ntae173_suppl_Supplementary_Data

## Data Availability

All data used in this article will be made available to readers by request. References 51–118 are available as Supplementary Material.
