# Supplementary material for: The Public Health Impact of a Ban on Flavored Cigars: A Decision-Theoretic Policy Framework
Source: Nicotine Tob Res. 2024 Jul 13;27(2):333–41. doi: 10.1093/ntr/ntae173 (PMC11750745; doi:10.1093/ntr/ntae173)
Supplement: ntae173_suppl_Supplementary_Data [file ntae173_suppl_supplementary_data.docx]

**Footnotes 51-118**

51. Hartmann-Boyce J, McRobbie H, Butler AR, et al. Electronic cigarettes for smoking cessation. *Cochrane Database Syst Rev.* 2021;9:CD010216.

52. Rostron BL, Chang JT, Chang CM, Jackson RA, Ambrose BK. ENDS Flavor Preference by Menthol Cigarette Smoking Status among US Adults, 2018-2019. *Int J Environ Res Public Health.* 2020;18(1).

53. Goldenson NI, Augustson EM, Shiffman S. Differences in switching away from cigarettes and JUUL use characteristics among adult menthol and nonmenthol smokers who purchased the JUUL System. *Drug Alcohol Depend.* 2022;231:109238.

54. Cook S, Hirschtick JL, Patel A, et al. A longitudinal study of menthol cigarette use and smoking cessation among adult smokers in the US: Assessing the roles of racial disparities and E-cigarette use. *Prev Med.* 2022;154:106882.

55. Levy DT, Cummings KM, Villanti AC, et al. A framework for evaluating the public health impact of e-cigarettes and other vaporized nicotine products. *Addiction.* 2017;112(1):8-17.

56. Levy DT, Cadham CJ, Li Y, et al. A Decision-Theoretic Public Health Framework for Heated Tobacco and Nicotine Vaping Products. *Int J Environ Res Public Health.* 2022;19(20).

57. Goniewicz ML, Gawron M, Smith DM, Peng M, Jacob P, 3rd, Benowitz NL. Exposure to Nicotine and Selected Toxicants in Cigarette Smokers Who Switched to Electronic Cigarettes: A Longitudinal Within-Subjects Observational Study. *Nicotine Tob Res.* 2017;19(2):160-167.

58. Xie W, Berlowitz JB, Raquib R, et al. Association of cigarette and electronic cigarette use patterns with all-cause mortality: A national cohort study of 145,390 US adults. *Prev Med.* 2024;182:107943.

59. Chaiton MO, Schwartz R, Tremblay G, Nugent R. Association of flavoured cigar regulations with wholesale tobacco volumes in Canada: an interrupted time series analysis. *Tob Control.* 2019;28(4):457-461.

60. Nguyen HV, Grootendorst P. Intended and unintended effects of restrictions on the sale of cigarillos to youth: evidence from Canada. *Tob Control.* 2015;24(4):382-388.

61. Villanti AC, Johnson AL, Ambrose BK, et al. Flavored Tobacco Product Use in Youth and Adults: Findings From the First Wave of the PATH Study (2013-2014). *Am J Prev Med.* 2017;53(2):139-151.

62. Villanti AC, Johnson AL, Glasser AM, et al. Association of Flavored Tobacco Use With Tobacco Initiation and Subsequent Use Among US Youth and Adults, 2013-2015. *JAMA Netw Open.* 2019;2(10):e1913804.

63. Villanti AC, Johnson AL, Halenar MJ, et al. Menthol and Mint Cigarettes and Cigars: Initiation and Progression in Youth, Young Adults and Adults in Waves 1-4 of the PATH Study, 2013-2017. *Nicotine Tob Res.* 2021;23(8):1318-1326.

64. Delnevo CD, Giovenco DP, Miller Lo EJ. Changes in the Mass-merchandise Cigar Market since the Tobacco Control Act. *Tob Regul Sci.* 2017;3(2 Suppl 1):S8-S16.

65. Courtemanche CJ, Palmer MK, Pesko MF. Influence of the Flavored Cigarette Ban on Adolescent Tobacco Use. *Am J Prev Med.* 2017;52(5):e139-e146.

66. Dai H, Leventhal AM. Prevalence of e-Cigarette Use Among Adults in the United States, 2014-2018. *JAMA.* 2019;322(18):1824-1827.

67. Levy DT, Tam J, Sanchez-Romero LM, et al. Public health implications of vaping in the USA: the smoking and vaping simulation model. *Popul Health Metr.* 2021;19(1):19.

68. Delnevo CD, Hrywna M. Clove cigar sales following the US flavoured cigarette ban. *Tob Control.* 2015;24(e4):e246-250.

69. Olson LT, Gammon DG, Rogers T, et al. Expanding local sales restrictions on flavoured tobacco products to include menthol: retail sales changes in two Minnesota cities. *Tob Control.* 2024;33(2):178-85.

70. Kotlyar M, Shanley R, Dufresne SR, Corcoran GA, Hatsukami DK. Effect on Tobacco Use and Subjective Measures of Including E-cigarettes in a Simulated Ban of Menthol in Combustible Cigarettes. *Nicotine Tob Res.* 2022;24(9):1448-1457.

71. Kotlyar M, Shanley R, Dufresne SR, Corcoran GA, Hatsukami DK. Effect of restricting menthol flavored cigarettes or E-cigarettes on smoking behavior in menthol smokers. *Prev Med.* 2022:107243.

72. Kotlyar M, Shanley R, Dufresne SR, et al. Effects on time to lapse of switching menthol smokers to non-menthol cigarettes prior to a cessation attempt: a pilot study. *Tob control.* 2021;30(5):574-577.

73. Kotlyar M, Shanley R, Dufresne SR, et al. Effects on Smoking Behavior of Switching Menthol Smokers to Non-menthol Cigarettes. *Nicotine Tob Res.* 2021;23(11):1921-1927.

74. Wackowski OA, Delnevo CD, Pearson JL. Switching to E-Cigarettes in the Event of a Menthol Cigarette Ban. *Nicotine Tob Res.* 2015;17(10):1286-1287.

75. Wackowski OA, Manderski MT, Delnevo CD. Young adults' behavioral intentions surrounding a potential menthol cigarette ban. *Nicotine Tob Res.* 2014;16(6):876-880.

76. Cadham CJ, Liber AC, Sanchez-Romero LM, et al. The actual and anticipated effects of restrictions on flavoured electronic nicotine delivery systems: a scoping review. *BMC Public Health.* 2022;22(1):2128.

77. Friedman A, Liber A, Crippen A, Pesko MF. E-cigarette Flavor Restrictions’ Effects on Tobacco Product Sales <https://papers.ssrn.com/sol3/papers.cfm?abstract_id=4586701>. Published 2023. Accessed January 6, 2024.

78. Satchell T, Diaz MC, Stephens D, Bertrand A, Schillo BA, Whitsel LP. The impact of two state-level approaches to restricting the sale of flavored tobacco products. *BMC Public Health.* 2022;22(1):1799.

79. Rogers T, Feld A, Gammon DG, et al. Changes in cigar sales following implementation of a local policy restricting sales of flavoured non-cigarette tobacco products. *Tob Control.* 2020;29(4):412-419.

80. Kingsley M, Setodji CM, Pane JD, et al. Longer-Term Impact of the Flavored Tobacco Restriction in Two Massachusetts Communities: A Mixed-Methods Study. *Nicotine Tob Res.* 2021;23(11):1928-1935.

81. Yang Y, Lindblom EN, Salloum RG, Ward KD. The impact of a comprehensive tobacco product flavor ban in San Francisco among young adults. *Addict Behav Rep.* 2020;11:100273.

82. Kingsley M, McGinnes H, Song G, Doane J, Henley P. Impact of Massachusetts' Statewide Sales Restriction on Flavored and Menthol Tobacco Products on Tobacco Sales in Massachusetts and Surrounding States, June 2020. *Am J Public Health.* 2022;112(8):1147-1150.

83. Edwards KC, Sharma E, Halenar MJ, et al. Longitudinal pathways of exclusive and polytobacco cigar use among youth, young adults and adults in the USA: findings from the PATH Study Waves 1-3 (2013-2016). *Tob Control.* 2020;29(Suppl 3):s163-s169.

84. Cohn A, Cobb CO, Niaura RS, Richardson A. The Other Combustible Products: Prevalence and Correlates of Little Cigar/Cigarillo Use Among Cigarette Smokers. *Nicotine Tob Res.* 2015;17(12):1473-1481.

85. Richardson A, Rath J, Ganz O, Xiao H, Vallone D. Primary and dual users of little cigars/cigarillos and large cigars: demographic and tobacco use profiles. *Nicotine Tob Res.* 2013;15(10):1729-1736.

86. Delnevo CD, Hrywna M, Giovenco DP, Miller Lo EJ, O'Connor RJ. Close, but no cigar: certain cigars are pseudo-cigarettes designed to evade regulation. *Tob Control.* 2017;26(3):349-354.

87. Delnevo CD, Hrywna M, Foulds J, Steinberg MB. Cigar use before and after a cigarette excise tax increase in New Jersey. *Addict Behav.* 2004;29(9):1799-1807.

88. Gammon DG, Loomis BR, Dench DL, King BA, Fulmer EB, Rogers T. Effect of price changes in little cigars and cigarettes on little cigar sales: USA, Q4 2011-Q4 2013. *Tob Control.* 2016;25(5):538-544.

89. Sharma E, Edwards KC, Halenar MJ, et al. Longitudinal pathways of exclusive and polytobacco smokeless use among youth, young adults and adults in the USA: findings from the PATH Study Waves 1-3 (2013-2016). *Tob Control.* 2020;29(Suppl 3):s170-s177.

90. Taylor KA, Sharma E, Edwards KC, et al. Longitudinal pathways of exclusive and polytobacco cigarette use among youth, young adults and adults in the USA: findings from the PATH Study Waves 1-3 (2013-2016). *Tob Control.* 2020;29(Suppl 3):s139-s146.

91. Rostron BL, Schroeder MJ, Ambrose BK. Dependence symptoms and cessation intentions among US adult daily cigarette, cigar, and e-cigarette users, 2012-2013. *BMC Public Health.* 2016;16(1):814.

92. Corey CG, Holder-Hayes E, Nguyen AB, et al. US Adult Cigar Smoking Patterns, Purchasing Behaviors, and Reasons for Use According to Cigar Type: Findings From the Population Assessment of Tobacco and Health (PATH) Study, 2013-2014. *Nicotine Tob Res.* 2018;20(12):1457-1466.

93. Mantey DS, Onyinye ON, Montgomery L. Prevalence and correlates of daily blunt use among U.S. African American, Hispanic, and White adults from 2014 to 2018. *Psychol Addict Behav.* 2021;35(5):514-522.

94. Watkins SL, Pieper F, Chaffee BW, Yerger VB, Ling PM, Max W. Flavored Tobacco Product Use Among Young Adults by Race and Ethnicity: Evidence From the Population Assessment of Tobacco and Health Study. *J Adolesc Health.* 2022;71(2):226-232.

95. Chen-Sankey JC, Choi K, Kirchner TR, Feldman RH, Butler J, 3rd, Mead EL. Flavored cigar smoking among African American young adult dual users: An ecological momentary assessment. *Drug Alcohol Depend.* 2019;196:79-85.

96. Giovenco DP, Spillane TE, Mauro CM, Martins SS. Cigarillo sales in legalized marijuana markets in the U.S. *Drug Alcohol Depend.* 2018;185:347-350.

97. Chu A, Chaiton M, Kaufman P, et al. Co-Use, Simultaneous Use, and Mixing of Cannabis and Tobacco: A Cross-National Comparison of Canada and the US by Cannabis Administration Type. *Int J Environ Res Public Health.* 2023;20(5).

98. Kostygina G, Ling PM. Tobacco industry use of flavourings to promote smokeless tobacco products. *Tob Control.* 2016;25(Suppl 2):ii40-ii49.

99. Food and Drug Administration. Tobacco Products Marketing Orders. PMTAs Web site. <https://www.fda.gov/tobacco-products/market-and-distribute-tobacco-product/tobacco-products-marketing-orders#Marketing%20Denial>. Published 2022. Accessed July 28, 2022.

100. Hrywna M, Lewis MJ, Ling PM. Nicotine Pouch Unit Sales in the US From 2016 to 2020. *JAMA.* 2021;326(22):2330-2331.

101. Majmundar A, Okitondo C, Xue A, Asare S, Bandi P, Nargis N. Nicotine Pouch Sales Trends in the US by Volume and Nicotine Concentration Levels From 2019 to 2022. *JAMA Netw Open.* 2022;5(11):e2242235.

102. Levy DT, Warner KE, Liber AC, et al. Potential Implications for Tobacco Industry Transformation of the Acquisition of Swedish Match by Philip Morris International. *Nicotine Tob Res.* 2023.

103. Rose SW, Ickes M, Patel M, et al. Centering equity in flavored tobacco ban policies: Implications for tobacco control researchers. *Prev Med.* 2022;165(Pt B):107173.

104. McGinnes H, Kingsley M, Song G, et al. Evaluation of a Statewide Flavored Tobacco Restriction on Use, Access, and Cessation Among Black and White Tobacco Users in Massachusetts. *Am J Health Promot.* 2023;37(7):905-914.

105. Farley SM, Sisti J, Jasek J, Schroth KRJ. Flavored Tobacco Sales Prohibition (2009) and Noncigarette Tobacco Products in Retail Stores (2017), New York City. *Am J Public Health.* 2020;110(5):725-730.

106. U.S. Department of Health and Human Services FaDA, Center for Tobacco Products. Scientific Assessment of the Impact of Flavors in Cigar Products. FDA. <https://www.fda.gov/media/157595/download>. Published 2022. Accessed September 25, 2023.

107. Andersen-Rodgers E, Zhang X, Vuong TD, et al. Are California's Local Flavored Tobacco Sales Restrictions Effective in Reducing the Retail Availability of Flavored Tobacco Products? A Multicomponent Evaluation. *Eval Rev.* 2021;45(3-4):134-165.

108. Vyas P, Ling P, Gordon B, et al. Compliance with San Francisco's flavoured tobacco sales prohibition. *Tob Control.* 2021;30(2):227-230.

109. Slater S, Pugach O, Rogers T, et al. Changes in Retail Tobacco Product Availability Following a Chicago City Ordinance Restricting Sales of Menthol and Other Flavored Tobacco Products Near Schools. *Health Educ Behav.* 2023;50(5):693-702.

110. Schroth KRJ, Kurti M, Delnevo CD. Flavored cigar availability in Oakland after a partial ban. *Addict Behav.* 2022;125:107150.

111. Jo CL, Williams RS, Ribisl KM. Tobacco products sold by Internet vendors following restrictions on flavors and light descriptors. *Nicotine Tob Res.* 2015;17(3):344-349.

112. Levy D, Chaloupka F, Lindblom E, et al. The US Cigarette Industry: An Economic and Marketing Perspective. *Tob Reg Sci.* 2019;5, (2):156-168.

113. Farley SM, Schroth KR, Grimshaw V, et al. Flavour chemicals in a sample of non-cigarette tobacco products without explicit flavour names sold in New York City in 2015. *Tob Control.* 2018;27(2):170-176.

114. Chaiton MO, Schwartz R, Cohen JE, Soule E, Zhang B, Eissenberg T. The use of flavour cards and other additives after a menthol ban in Canada. *Tob Control.* 2021;30(5):601-602.

115. Kyriakos CN, Qi D, Chang K, Laverty AA, Filippidis FT. Global market trends of flavor capsule cigarettes and menthol (non-capsule) cigarettes: An ecological analysis using commercial data across 78 countries, 2010-2020. *Tob Induc Dis.* 2022;20:85.

116. Moodie C, Thrasher JF, Barnoya J, et al. Tobacco industry claims about transformation are inconsistent with combustible cigarette innovations: The case of flavour capsule cigarettes. *Nicotine Tob Res.* 2023;25(12):1891-1895.

117. Thrasher JF, Abad-Vivero EN, Moodie C, et al. Cigarette brands with flavour capsules in the filter: trends in use and brand perceptions among smokers in the USA, Mexico and Australia, 2012-2014. *Tob Control.* 2016;25(3):275-283.

118. Schroth KRJ, Delnevo CD, Villanti AC. Closing the loopholes on a flavored cigar ban: Anticipated challenges and solutions. *Prev Med.* 2022;165(Pt B):107197.
